# The Apoptogenic Toxin AIP56 Is Secreted by the Type II Secretion System of *Photobacterium damselae* subsp. *piscicida*

**DOI:** 10.3390/toxins9110368

**Published:** 2017-11-14

**Authors:** Ana do Vale, Cassilda Pereira, Carlos R. Osorio, Nuno M. S. dos Santos

**Affiliations:** 1 Fish Immunology and Vaccinology Group, IBMC-Instituto de Biologia Molecular e Celular, Universidade do Porto, 4200-135 Porto, Portugal; cassilda.pereira@i3s.up.pt (C.P.); nsantos@ibmc.up.pt (N.M.S.d.S.); 2 i3S-Instituto de Investigação e Inovação em Saúde, Universidade do Porto, 4200-135 Porto, Portugal; 3 Departamento de Microbioloxía e Parasitoloxía, Instituto de Acuicultura, Universidade de Santiago de Compostela, 15782 Santiago de Compostela, Spain; cr.osorio@usc.es

**Keywords:** exotoxin, secretion, T2SS, polar localisation

## Abstract

AIP56 (apoptosis-inducing protein of 56 kDa) is a key virulence factor of *Photobacterium damselae* subsp. *piscicida (Phdp),* the causative agent of a septicaemia affecting warm water marine fish species. *Phdp*-associated pathology is triggered by AIP56, a short trip AB toxin with a metalloprotease A domain that cleaves the p65 subunit of NF-κB, an evolutionarily conserved transcription factor that regulates the expression of inflammatory and anti-apoptotic genes and plays a central role in host responses to infection. During infection by *Phdp*, AIP56 is systemically disseminated and induces apoptosis of macrophages and neutrophils, compromising the host phagocytic defence and contributing to the genesis of pathology. Although it is well established that the secretion of AIP56 is crucial for *Phdp* pathogenicity, the protein secretion systems operating in *Phdp* and the mechanism responsible for the extracellular release of the toxin remain unknown. Here, we report that *Phdp* encodes a type II secretion system (T2SS) and show that mutation of the EpsL component of this system impairs AIP56 secretion. This work demonstrates that *Phdp* has a functional T2SS that mediates secretion of its key virulence factor AIP56.

## 1. Introduction

*Photobacterium damselae* subsp. *piscicida* (*Phdp*) is a Gram-negative bacterium that causes systemic acute infections with very high mortalities in several wild and cultured marine fish species (reviewed in [1,2,3]). *Phdp* infections are characterised by the occurrence of generalised bacteraemia and pronounced cytopathology with disseminated tissue necrosis [4]. It is now well established that the *Phdp*-associated pathology is triggered by AIP56 (apoptosis inducing protein of 56 kDa), a plasmid-encoded toxin secreted by virulent *Phdp* strains [5]. Genes coding for AIP56 homologues are present in different organisms, mainly marine *Vibrio* species and *Arsenophonus nasoniae*. It has been shown that AIP56 is an AB-type toxin, with a catalytic A domain at its N-terminus and a B domain that mediates binding/internalization into susceptible cells at its C-terminal region [6]. The catalytic domain of AIP56 displays zinc-dependent metalloprotease activity against the p65 subunit of NF-κB [6], an evolutionarily conserved transcription factor that plays crucial roles in host responses to invasion by pathogenic microorganisms by regulating the expression of inflammatory and anti-apoptotic genes.

In early phases of *Phdp* infection, when multiplication of *Phdp* becomes detectable in infected tissues, pronounced infiltration of macrophages and neutrophils occurs [4]. With the progress of the infection, the pathogen disseminates and multiplies extensively and AIP56 is detected in the systemic circulation [4]. The presence of circulating toxin triggers the apoptotic destruction of macrophages and neutrophils and explains the phagocyte depletion observed in advanced *Phdp* infections [4]. The neutralization of the host phagocytic defence by AIP56 allows survival of the pathogen and its unrestricted extracellular multiplication, contributing to the fatal outcome of *Phdp* infections. The marked reduction on the number of phagocytes, by compromising the host capacity to clear apoptotic cells, leading to the lysis of the phagocytes by post-apoptotic secondary necrosis with consequent release of their highly cytotoxic, tissue-damaging contents [4,7], also contributes to the cytopathology observed in *Phdp*-infected fish. Although it is well established that AIP56 plays a central role in the establishment of *Phdp* infection and in the development of the infection-associated pathology, the protein secretion systems operating in *Phdp* and the mechanism responsible for the extracellular release of the toxin are completely unknown.

Gram-negative bacteria have developed dedicated systems for extracellular protein secretion (reviewed in [8]). Amongst them, the T2SS, which is widely distributed in Gram-negative species, is responsible for secreting a large number of proteins across the outer membrane (reviewed in [9,10]). Substrates secreted via the T2SS include key virulence factors and degradative enzymes, conferring to this system a major survival role for pathogenic and environmental species. Examples of virulence-associated T2SS substrates include cholera toxin (reviewed in [11]) and *P. aeruginosa* exotoxin A [12]. The T2SS consists of 12–15 different proteins and spans from the cytoplasm, via the inner membrane and the periplasm, to the actual pore in the outer membrane (reviewed in [10,13,14]). It comprises four subassemblies: an outer membrane secretin channel, a periplasmic pseudopilus, an inner membrane platform and an associated cytoplasmic ATPase [14]. Type II-dependent cargos are first translocated via the general secretory (Sec) pathway or the twin-arginine transport (Tat) system into the periplasm, where they acquire the correct tertiary and/or quaternary structure before being translocated in their native folded state across the outer membrane through the T2SS [14]. In this work, we demonstrate that *Phdp* has a functional type II secretion system (T2SS) that mediates the secretion of AIP56 into the extracellular environment.

## 2. Results and Discussion

**Figure 1:**
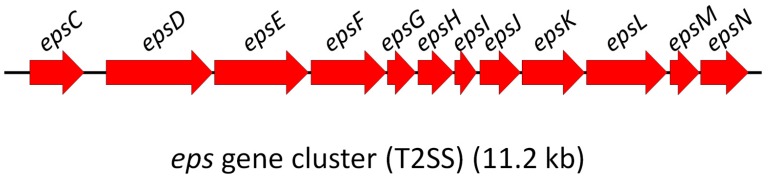
*P. damselae* subsp. *piscicida* encodes a T2SS. Physical map of the *P. damselae* subsp. *piscicida* MT1415 12-gene cluster encoding the T2SS.

Recently, it has been shown that *P. damselae* subsp. *damselae* (*Phdd*) harbors a T2SS that mediates the secretion of its major virulence factors Dly, plasmid-encoded HlyA (PhlyP), and chromosome-encoded HlyA (PhlyC) [15]. This prompted us to investigate if *P. damselae* subsp. *piscicida* (*Phdp*) also encoded a T2SS. Here, we show that, indeed, *Phdp* harbours T2SS genes highly identical to the genes reported in *Phdd*. The complete nucleotide sequence of the *eps* (extracellular protein secretion) gene cluster encoding the T2SS in *Phdp* MT1415 was established as a 12-gene operon which covered a 11,233 bp region spanning from the *epsC* start codon to the *epsN* stop codon (Figure 1).

The amino acid sequence of the 12 Eps proteins was found to be highly conserved among MT1415 isolate and other subspecies *piscicida* strains (Table 1). Indeed, the sequence comparison analyses revealed that 11 of the MT1415 Eps proteins are >95% identical to the subspecies *damselae* Eps proteins with the exception of EpsC, the first protein of the cluster, which is only 73% identical between the two subspecies (Table 1). EpsC is an inner membrane component of the T2SS and interacts with EpsD, an outer membrane secretin that forms a multimeric pore for the translocation of the secreted proteins [16]. The reason why EpsC protein has diverged so much in these two closely related subspecies is currently unknown.

**Figure 2:**
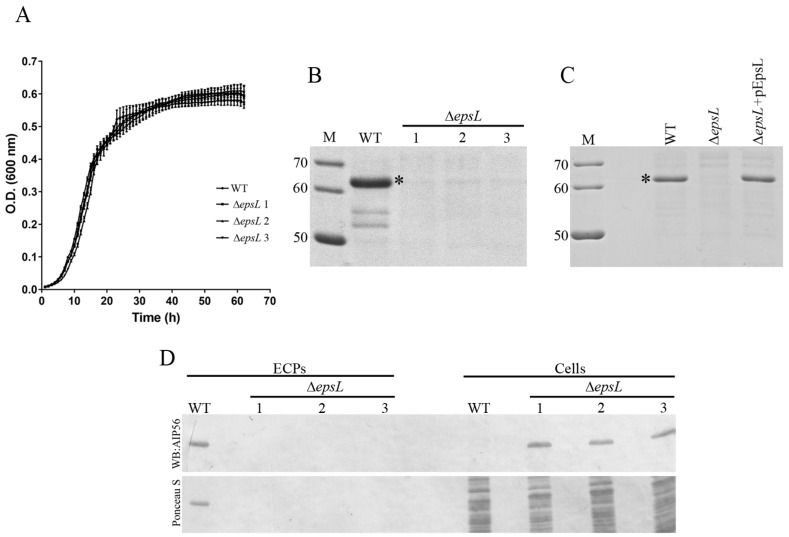
Deletion of *epsL* impairs AIP56 secretion. (**A**) Growth curves of wild type (WT) or isogenic Δ*epsL* mutant strain grown in TSB-1 at 25 °C. The curves were generated from three replicates for each strain. (**B**,**C**) Secreted protein profiles of WT and Δ*epsL* strain. SDS-PAGE analysis of culture supernatants from WT, Δ*epsL* (three independent clones) and pEpsL complemented strains. Bacterial strains were grown in TSB-1 at 25 °C to an OD 600 nm of approximately 1 or 0.35 for (**B**,**C**), respectively. Supernatants (ECPs) were collected, filtered and subjected to TCA precipitation. TCA precipitates were dissolved in running buffer and equivalents to 0.3 or 1.5 mL cultures (for (**B**,**C**), respectively) were subjected to SDS-PAGE. The gel was stained with Coomassie-blue. Numbers at the left indicate the molecular weight of the markers (M) in kDa. Asterisks indicate the AIP56 band. Data are representative of three independent experiments. (**D**) In *epsL* deleted strains, AIP56 is synthesised but retained inside the cells. WT and Δ*epsL* strains were grown in TSB-1 at 25 °C to an OD 600 nm of approximately 1 (exponential phase). ECPs were collected, filtered and subjected to TCA precipitation. AIP56 in the bacterial pellets and ECPs was detected by western blotting. Upper panel: western blotting (AIP56). Lower panel: total protein loading (Ponceau S).

AIP56 is synthesised as a precursor protein with a typical Sec signal peptide at the *N*-terminus suggesting that it is secreted by a two-step process that begins with Sec-dependent translocation across the cytoplasmic membrane into the periplasm [5]. Since many proteins that are secreted via Sec system into the periplasm are then transported across the outer membrane through the T2SS, we hypothesised that this system could be responsible for the secretion of AIP56. To test this hypothesis, we generated strains lacking the *epsL* gene, which encodes an inner membrane-spanning protein known to establish a critical link between the cytoplasmic and the periplasmic part of the T2SS [17] and analysed if the secretion of AIP56 was affected in Δ*epsL* strains. To exclude off-target effects, three independent Δ*epsL* clones were studied. No differences in growth between the WT and Δ*epsL* strains were observed (Figure 2A). SDS-PAGE analysis of culture supernatants revealed that secretion of AIP56 was abolished in the *epsL* mutants (Figure 2B) and that complementation with plasmid-encoded EpsL completely restored AIP56 secretion (Figure 2C). The absence of AIP56 in the Δ*epsL* supernatants was not due to decreased synthesis of the toxin, but rather to the inability of the mutant to export the protein to the extracellular milieu, as concluded from western blotting analysis which showed that the mutant produced amounts of AIP56 similar to the amount produced by the WT strain (Figure 2D).

**Figure 3:**
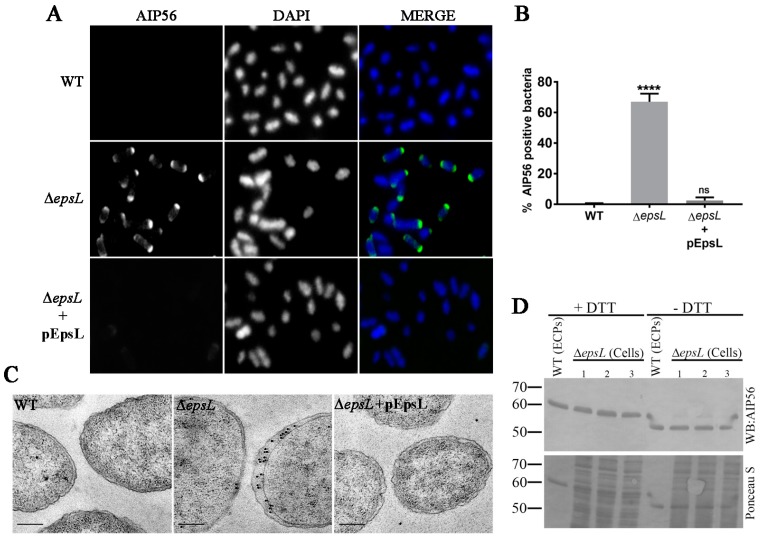
In Δ*epsL* mutants, AIP56 accumulates in the periplasm. (**A**) Visualization of AIP56 localization in WT, Δ*epsL* and pEpsL complemented strains by fluorescence microscopy. Labelling was performed with an anti-AIP56^286–497^ rabbit antibody and the anti-rabbit secondary antibody was conjugated to Alexa-488 (green on the coloured panels). The cells were counterstained with DAPI (blue in the coloured panels). (**B**) Results of the quantification of AIP56 positive cells in the immunofluorescence micrographs of WT, Δ*epsL* and Δ*epsL* + pEpsL strains. A minimum of 600 cells per condition were counted in each experiment, using Fiji software. Values are mean ± SD of two independent experiments. One-way ANOVA followed by Tukey post hoc test, **** *p* < 0.0001. (**C**) Transmission electron micrographs of ultrathin sections of WT and Δ*epsL* cells stained with an anti-AIP56^286–497^ rabbit antibody followed by a gold-conjugated secondary antibody (15 nm gold). Bar = 200 nm. (**D**) Western blotting analysis of AIP56 in the WT ECPs and Δ*epsL* cells under reducing (+DTT) and non-reducing (−DTT) conditions. Samples equivalent to 75 μL initial culture were loaded in each lane. Upper panel: western blotting (AIP56). Lower panel: total protein loading (Ponceau S). Numbers at the left indicate the position and molecular weight of the markers (in kDa).

Immunofluorescence microscopy was used to determine the subcellular localization of AIP56 in Δ*epsL* cells. The results confirmed the accumulation of AIP56 in *epsL*-deleted cells (Figure 3A,B), and revealed that in these cells, the toxin presents a peripheral distribution (consistent with localization at the periplasm) with marked polar accumulation, with one of the poles staining much brighter than the other (Figure 3A). As expected, complementation with plasmid-encoded EpsL resulted in a phenotype similar to the one observed for the wild type strain (Figure 3A,B). The periplasmic localization of AIP56 as well as its polar accumulation in Δ*epsL* cells was confirmed by immunogold-labelling coupled with transmission electron microscopy (Figure 3C).

Thus far, the reasons for the polar localization of AIP56 in the periplasm of *Phdp* upon T2SS disruption remain to be investigated. One possibility is that localization of the toxin results from polarized Sec-secretion into the periplasm followed by accumulation at the secretion site. Alternatively, it may result from redistribution and polar retention of AIP56 that has been randomly transported across the cytoplasmic membrane and retained at the periplasm. Apart from determining which of these alternatives is occurring in this context, it would be important to study the distribution of the T2SS apparatus in *Phdp* and to investigate if secretion of AIP56 by these bacteria occurs at the poles. The polar localization of some bacterial secretion systems has been reported, including for the SPI2-encoded T3SS of Salmonella [18] and several T4SS [19,20,21,22] but has never been reported in the case of the T2SS. In fact, studies in *Vibrio cholerae* showed that when produced at endogenous levels, the T2SS components EpsM, EspG and EpsC localize at distinct foci distributed around the cell periphery [23] and similar distribution pattern has been reported for the *Klebsiella oxytoca* PulL and PulM (EpsL and EspM homologues, respectively) [24].

As discussed above, proteins secreted by the T2SS are first translocated via the Sec or Tat systems across the cytoplasmic membrane into the periplasm (reviewed in [25]). The Sec pathway primarily translocates proteins in their unfolded state, whereas the Tat pathway secretes folded proteins (reviewed in [25,26]). It has been previously shown that AIP56 contains two cysteine residues which form an intramolecular disulphide bond [6] that is required for toxicity. Western blotting analysis under reducing and non-reducing conditions revealed that the AIP56 recovered from the periplasm of the Δ*epsL* mutant and the AIP56 obtained from the wild type culture supernatants have a similar electrophoretic behaviour (Figure 3D). The higher electrophoretic mobility observed under non-reducing conditions confirmed the presence of the intramolecular disulphide bridge, suggesting that upon secretion into the periplasm and before secretion across the outer membrane AIP56 folds through a process that involves formation of the disulphide bond.

In conclusion, we have shown that *Phdp* has a functional T2SS and that this system is required for secretion of its key virulence factor AIP56. Accordingly, disruption of the T2SS blocks export of AIP56 from the periplasm to the extracellular medium, resulting in the periplasmic polar accumulation of AIP56. Whether this polar localization of the AIP56 transported into the periplasm has any functional significance or is a consequence of the accumulation of high amounts of toxin at the periplasmic compartment remains to be elucidated.

## 3. Materials and Methods

### 3.1. Reagents

Tryptic soy broth (TSB) and tryptic soy agar (TSA) were from Difco^TM^ (Becton, Dickinson and Company, Franklin Lakes, NJ, USA) The rabbit antibodies against full length AIP56 or AIP56^286–497^ were produced at PickCell Laboratories (Amsterdam, The Netherlands) or Davids Biotechnologie (Regensburg, Germany) using recombinant AIP56 or AIP56^286–497^ as antigens. Recombinant proteins used for immunizations were produced and purified as previously described [6]. Anti-AIP56 and anti-AIP56^286–497^ IgG fractions were affinity-purified from the immune sera at the producing companies using immobilized AIP56 or AIP56^286–497^ as ligands. Goat anti-rabbit alkaline phosphatase-conjugated secondary antibody (A9919) was from Sigma-Aldrich (St. Louis, MO, USA). Alexa Fluor 488 goat anti-rabbit IgG (heavy plus light chain) was from Molecular Probes (Eugene, OR, USA).

### 3.2. Bacterial Strains and Culture Conditions

*P. damselae* subsp. *piscicida* virulent strain MT1415 isolated from sea bass in Italy [5] was routinely cultured at 25 °C in TSB or TSA supplemented with NaCl to a final concentration of 1% (*w*/*v*) (TSB-1 and TSA-1, respectively). Stocks of bacteria were maintained at −80 °C in TSB-1 supplemented with 15% (*v*/*v*) glycerol. To obtain growth curves and samples for electron microscopy, bacteria grown on TSA-1 plates for 48 h were suspended in TSB-1 at an OD 600 nm of 0.5–0.6. These suspensions were used to inoculate 20 mL TSB-1, at 1:100 dilutions. For growth curves, one mL aliquots were removed (in triplicate), transferred to wells of a 24-well culture plate and the OD 600 nm determined kinetically using BioTek Synergy 2 spectrofluorometer (BioTeK U.S., Winooski, VT, USA) at 25 °C with continuous slow agitation, for 60–70 h at a frequency of 1 point/h. The curves were constructed using GraphPad Prism software version 7.03 (La Jolla, CA, USA). For electron microscopy, the 20 mL cultures were incubated at 25 °C with shaking until reaching an OD 600 nm of 0.7–0.8 (exponential phase), centrifuged (6000× *g*, 5 min, 4 °C) and the bacterial pellet processed as described below. For immunofluorescence microscopy, bacteria grown on TSA-1 plates for 8 h were resuspended in TSB-1 at an OD 600 nm of 0.5–0.6. These suspensions were used to inoculate 20 mL of TSB-1 at 1:20 dilution and cultures were grown at 25 °C with shaking until reaching an OD 600 nm of 0.35–0.5. Bacterial suspensions were centrifuged (6000× *g*, 5 min, 4 °C) and the bacterial pellet processed for immunofluorescence as described below.

### 3.3. T2SS Gene Cluster Sequencing

To date, the only characterised T2SS system in a member of the *Photobacteriaceae* is that of *P. damselae* subsp. *damselae* RM71 [15]. To obtain the complete sequence of the subspecies *piscicida* MT1415 T2SS gene cluster, a collection of PCR primers were designed on the basis of the subspecies *damselae* RM71 T2SS gene cluster sequence (GenBank Accession Number LYBT00000000). The complete sequence of the MT1415 T2SS gene cluster (*eps* genes) was obtained by PCR amplification and DNA sequencing by primer walking. This sequence has been deposited in GenBank database under Accession Number MF537591. The MT1415 Eps sequences were then compared to the correspondent sequences retrieved from the complete genome sequences available at Genebank for *Phdp* strain L091106-03H (Accession number MCFX00000000), *Phdp* strain DI21 (Accession number AKYG00000000), *Phdp* strain OT-51443(Accession number BDMQ01000000), *Phdd* strain CIP 102761 (Accession number NZ_ADBS01000000) and *Vibrio cholerae* N16961 (Accession numbers AE003852 and AE003853).

### 3.4. Mutant Construction

To functionally characterise the role of the T2SS in AIP56 toxin secretion, the *epsL* gene of *P. damselae* subsp. *piscicida* MT1415 was selected to construct an in-frame deletion mutant. First, a spontaneous rifampicin (Rf^R^) resistant mutant of MT1415 was selected, to aid in the counter selection after conjugation experiments. A nonpolar deletion of *epsL* was constructed in MT1415-Rf^R^ by PCR amplification of the DNA flanking the amino- and carboxy-termini of the gene. Primers used for these amplifications were: MT1415-epsL-mut-1: GCTCTAGAGATGTGAAATTGCTAGAGCC (*Xba*I site underlined); MT1415-epsL-mut-2: GCGGATCCCTACTCAGCCTTATCGTCAG (*Bam*HI site underlined); MT1415-epsL-mut-3: GCGGATCCGCCGAGTAAATGGTGCCTTT (*Bam*HI site underlined); and MT1415-epsL-mut-4: GCGAATTCCAGCTTCGCGCCCACTTGGC (*Eco*RI site underlined). These two PCR fragments were subsequently fused together, and this resulted in an in-frame deletion of approximately 90% of the *epsL* coding sequence. Hi-Fidelity Kapa *Taq* (Kapa Biosystems, Wilmington, MA, USA)) was used for all PCR amplifications. Allelic exchange was basically performed as described previously [15]. The suicide vector pNidKan contains a *Km^r^* gene and the *sacB* gene conferring sucrose sensitivity. In addition, this suicide vector contains a R6K *ori*, which depends on the product of the *pir* gene for replication. The plasmid construction harbouring the deleted allele was transferred from *E. coli* S17-1-λ*pir* into the rifampicin-resistant derivative MT1415-Rf^R^. After conjugation for 24 h on TSA plates prepared with seawater, cells were recovered from the plate, re-suspended in TSB-1, and spread on TSA-1 plates, selecting for rifampicin resistance (selects MT1415) and for kanamycin resistance (selects for plasmid integration). A selected first cross-over clone was subsequently selected for sucrose resistance (15% *w*/*v*) for a second event of recombination. This led to *P*. *damselae* subsp. *piscicida* mutant strain MT1415-Rf^R^ Δ*epsL*. PCR amplification and further DNA sequencing of the region involved in the mutagenesis confirmed the presence of the correct mutant allele. Three independently obtained Δ*epsL* mutants were selected for the functional studies.

### 3.5. Gene Complementation

The *epsL* gene is the 10th gene within the *epsL* operon and lacks its own promoter, being likely transcribed from a promoter located upstream of *epsC*. Therefore, for complementation of *epsL* mutants, we followed a strategy that consisted of cloning the complete coding sequence of *epsL* gene downstream of the promoter of the *P. damselae* subsp. *damselae* haemolysin gene *hlyA_ch_*, a promoter that in previous studies proved to be expressed under standard culture conditions [27]. The *hlyA_ch_* promoter fused to the *epsL* gene was then cloned into the mobilizable vector pBBR1MCS5. The resulting plasmid construction, pEpsL, was mobilized from *E. coli* S17-1-λ*pir* into the MT1415-Rf^R^ *epsL* mutants.

### 3.6. Preparation of Extracellular Products

To prepare extracellular products (ECPs), bacteria were grown in TSB-1 at 25 °C with shaking (100 rpm) to the indicated optical density (OD) at 600 nm. Bacterial suspensions were centrifuged (6000× *g*, 5 min, 4 °C), the bacterial cell pellet stored and the culture supernatants collected and filtered through 0.22 μm-pore-size filter (Schleicher & Shuell, Dassel, Germany). For SDS-PAGE, proteins from 1.5 mL cell-free culture supernatant were precipitated with 10% (*w*/*v*) TCA for 30 min on ice and recovered by centrifugation. Protein pellets were washed in 10% (*w*/*v*) TCA followed by a washing in acetone, and air-dried.

### 3.7. SDS-PAGE and Western Blotting

Bacterial cell pellets and precipitated ECPs were solubilized in SDS-sample buffer (50 mM Tris-HCl (pH 8.8), 2% SDS, 0.05% bromophenol blue, 10% glycerol, 2 mM EDTA, and 100 mM DTT) and subjected to SDS-PAGE in 10 or 12% polyacrylamide gels using the Laemmli discontinuous buffer system [28]. Proteins in the gels were stained with Coomassie Brilliant Blue or electro blotted onto Amersham^TM^ Protran^TM^ 0.45 μm nitrocellulose membranes (GE Healthcare Life science, Buckinghamshire, UK), according to the manufacturer’s instructions. The efficiency of transfer and the protein loading on the membranes were controlled by staining with Ponceau S. The membranes were blocked for 1 h at RT with 5% skim milk in Tris-buffered saline (TBS) containing 0.1% Tween 20 (TBS-T) followed by incubation for 1h at RT with the anti-AIP56 rabbit antibody diluted in blocking buffer. Rabbit IgGs on western-blots were detected with a sheep anti-rabbit alkaline phosphatase conjugated secondary antibody (AP311, The binding Site) followed by nitroblue tetrazolium-5-bromo-4-chloro-3-indolylphosphate (NBT/BCIP) (Promega, Madison, WI, USA) development. Blots presented show representative results from at least three independent experiments.

### 3.8. Immunofluorescence Microscopy

Staining for immunofluorescence microscopy was performed on bacterial cells in suspension, at RT. Cells were fixed with ice-cold 4% (*w*/*v*) paraformaldehyde in PBS for 30 min, permeabilized with 0.1% Triton X-100 in PBS for 45 min followed by incubation in PBS containing 100 μg mL^−1^ freshly prepared lysozyme and 5 mM EDTA for 45 min with gentle agitation. Blocking was in 1% BSA in PBS for 1 h followed by incubation with the anti-AIP56 rabbit antibody in blocking buffer for 1 h in a humidified chamber. The cells were incubated with secondary antibody diluted in blocking buffer for 1 h followed by incubation with 0.5 μg mL^−1^ 4′,6-diamidino-2-phenylindole (DAPI). A small volume of the bacterial suspension was dropped on a coverslip, allowed to dry at 37 °C and mounted with VectaShield (Vector Laboratories, Burlingame, CA, USA) on a glass slide (bacterial-side-down). The preparations were observed with a PlanApo 100x/1.49 TIRF objective in a Nikon TiE microscope (Nikon Instruments Inc., Melville, NY, USA) controlled with Nikon NIS elements software version 5.00 (Nikon Instruments Inc., Melville, NY, USA) imaged with an Andor iXon 888 EMCCD camera (Andor Technology Ltd., Belfast, UK) and processed using the Fiji software (ImageJ version 1.51n, NIH, USA) [29].

### 3.9. Transmission Electron Microscopy

For electron microscopy, samples were fixed in 0.05% glutaraldehyde, 2% paraformaldehyde, 10 mM calcium chloride for 24 h and post-fixed in 1% OsO4-10 mM calcium chloride for 16–24 h followed by 1% uranyl acetate for 1 h. All incubations were done at RT. The samples were then embedded in Epon resin after dehydration in a graded series of ethanol. Ultrathin sections (40–60 nm thickness) were prepared on a RMC Ultramicrotome (PowerTome, Tucson, AZ, USA) using diamond knifes (DDK, Wilmington, DE, USA). For immuno-detection of AIP56, grids were washed in tris buffered saline (TBS), incubated in antigen retrieval solution (14.4% sodium metaperiodate) for 1 h, washed for 10 min in TBS, and incubated for 30 min in in TBS containing 2% BSA for blocking free protein-binding sites. Grids were then incubated with the anti-AIP56^286–497^ antibody diluted 1:1000 in TBS with 2% BSA, washed four times in TBS with 0.1% BSA and then incubated with the secondary antibody (goat anti-rabbit IgG, conjugated with 15 nm gold), diluted 1:20 in TBS with 1% BSA. After 45 min, the grids were washed four times with TBS and fixed in 1% glutaraldehyde for 5 min. Finally, samples were washed six times with MilliQ water (Millipore, Billerica, MA, USA) for 10 min, air-dried and contrasted with uranyl acetate (saturated aqueous solution) for 5 min. Observations were done under a JEOL JEM 1400 TEM (Tokyo, Japan) and images were digitally recorded using a CCD digital camera Orius 1100 W (Gatan Inc., Pleasanton, CA, USA).

## Figures and Tables

**Table 1:** Identity between the T2SS proteins of *Photobacterium damselae* subsp. *piscicida* MT1415 and related strains and species. Codes between brackets indicate the analysed strains. The per cent identity numbers refer to the amino acid sequences of the predicted T2SS proteins.

		Identity (%)			
Protein	# Residues	*P. damselae* subsp. *piscicida* (DI21 and L091106-03H)	*P. damselae* subsp. *piscicida* (OT-51443)	*P. damselae* subsp. *damselae* (CIP 102761)	*V. cholerae* (N16961)
**EpsC**	298	100	99	73	43
**EpsD**	668	100	100	97	66
**EpsE**	502	100	100	99	80
**EpsF**	406	100	100	99	68
**EpsG**	146	100	100	95	81
**EpsH**	193	100	100	95	45
**EpsI**	120	100	100	100	60
**EpsJ**	218	100	99	95	50
**EpsK**	334	100	99	96	53
**EpsL**	437	100	99	96	41
**EpsM**	159	100	100	99	47
**EpsN**	253	100	99	98	43
